# Deep learning-based end-to-end automated stenosis classification and localization on catheter coronary angiography

**DOI:** 10.3389/fcvm.2023.944135

**Published:** 2023-02-07

**Authors:** Chao Cong, Yoko Kato, Henrique Doria De Vasconcellos, Mohammad R. Ostovaneh, Joao A. C. Lima, Bharath Ambale-Venkatesh

**Affiliations:** ^1^Division of Cardiology, Johns Hopkins University, Baltimore, MD, United States; ^2^School of Electrical and Electronic Engineering, Chongqing University of Technology, Chongqing, China; ^3^Division of Radiology, Johns Hopkins University, Baltimore, MD, United States

**Keywords:** stenosis localization, stenosis classification, catheter coronary angiography, end-to-end workflow, deep learning, redundancy training

## Abstract

**Background:**

Automatic coronary angiography (CAG) assessment may help in faster screening and diagnosis of stenosis in patients with atherosclerotic disease. We aimed to provide an end-to-end workflow that separates cases with normal or mild stenoses from those with higher stenosis severities to facilitate safety screening of a large volume of the CAG images.

**Methods:**

A deep learning-based end-to-end workflow was employed as follows: (1) Candidate frame selection from CAG videograms with Convolutional Neural Network (CNN) + Long Short Term Memory (LSTM) network, (2) Stenosis classification with Inception-v3 using 2 or 3 categories (<25%, >25%, and/or total occlusion) with and without redundancy training, and (3) Stenosis localization with two methods of class activation map (CAM) and anchor-based feature pyramid network (FPN). Overall 13,744 frames from 230 studies were used for the stenosis classification training and fourfold cross-validation for image-, artery-, and per-patient-level. For the stenosis localization training and fourfold cross-validation, 690 images with > 25% stenosis were used.

**Results:**

Our model achieved an accuracy of 0.85, sensitivity of 0.96, and AUC of 0.86 in per-patient level stenosis classification. Redundancy training was effective to improve classification performance. Stenosis position localization was adequate with better quantitative results in anchor-based FPN model, achieving global-sensitivity for left coronary artery (LCA) and right coronary artery (RCA) of 0.68 and 0.70.

**Conclusion:**

We demonstrated a fully automatic end-to-end deep learning-based workflow that eliminates the vessel extraction and segmentation step in coronary artery stenosis classification and localization on CAG images. This tool may be useful to facilitate safety screening in high-volume centers and in clinical trial settings.

## Introduction

Coronary artery disease (CAD) is the leading cause of morbidity and mortality worldwide (1). X-ray coronary angiography (CAG) is the current gold standard imaging technique for CAD diagnosis. Expert CAG interpretation requires considerable “hands-on” training both visually and cognitively. In clinical practice and also for quality control purposes in research settings, screening CAG studies visually to distinguish cases with normal or mild stenosis from those with higher stenosis severity is a time-consuming process even for experienced readers. Developing an automatic CAG assessment tool to exclude normal or mild stenosis cases would facilitate diagnosis and treatment and enable the screening of large data sets for quality control purposes.

Recent studies confirmed the feasibility of using deep learning methods for CAG stenosis detection. Generally, the method consists of multiple steps. The most widely used vessel-based workflow starts from the visual or automatic selection of candidate frames (2–4) or regions (5, 6) from a CAG video. This is followed by the artery extraction using image segmentation algorithms (7) like center-tracking (8, 9), model-based (10), or Convolutional Neural Network (CNN) (11–15). Finally, individual stenotic lesion localization and classification is performed in two ways: patch-wise (16–18) and image-wise (2, 3, 6).

However, there are limitations in previous CAG stenosis classification and detection methods. One of the main drawbacks is that the vessel shape and characterization (19, 20) were not well exploited from a multi-view CAG study, causing a relatively low accuracy in detecting the stenotic lesions, especially in curved or bifurcation regions in the vascular tree (21, 22). Another limitation is that there are numerous pre-processing stages (manually or automatically) in some methods (15, 18, 23), such as detecting keyframes/region/views from a CAG sequence, or annotating segmentation for vessels, or preparing patches and labels for training procedure. The need for extensive human interaction during image data and training label preparation, in addition to addressing problems of sampling imbalance during supervised-learning, has led to algorithms that are commonly evaluated on small datasets prone to overfitting (7). Clinically speaking, those studies generally aimed to differentiate significant stenosis from non-significant stenosis in CAG images while developing a tool to facilitate safety screening of a large volume of CAG images by separating cases with normal or mild stenoses from those with higher stenosis severities have not been targeted (24).

In this study, we propose a fully automatic, deep learning-based end-to-end CAG stenosis detection method to achieve efficient safety screening and precise localization of stenoses. Our method consists of following unique steps that (1) it eliminates the vessel extraction and segmentation step for supervised learning; (2) the CNN + LSTM structure is designed for automatic detection of candidate frames from CAG sequences to improve training efficiency and reduce overfitting; (3) a multi-view analyzing architecture is established to train CNNs for different angle-views and generate classification results in artery-level and patient-level; (4) the redundancy training strategy is proposed to eliminate the negative effect of background and unnecessary features in training; and (5) the unsupervised- and supervised-learning methods are explored to localize the coronary stenoses in CAG images, which includes an anchor-based feature pyramid network (FPN).

## Materials and methods

### Study population

This research was retrospectively performed on 230 participants with available data from a ‘‘Combined Non-invasive Coronary Angiography and Myocardial Perfusion Imaging Using 320 Detector Computed Tomography (CORE320)’’ study (NCT00934037),^1^ a prospective, multicenter, international study that assessed the performance of combined 320-row CTA and myocardial CT perfusion imaging (CTP) in comparison with the combination of invasive CAG and single-photon emission computed tomography myocardial perfusion imaging (SPECT-MPI) for detecting myocardial perfusion defects and luminal stenosis in patients with suspected CAD (25, 26). For the stenosis classification, 36 studies out of 230 were excluded from the training due to the low image quality or contrasting condition. These images, however, were included for evaluation. The original CORE320 study was approved by central and local institutional review boards, and written informed consent was obtained from all participants (25, 26). Given the retrospective and ancillary nature of the data, the current study is covered by the original CORE320 study IRB.

### Candidate frame selection

**FIGURE 1:**
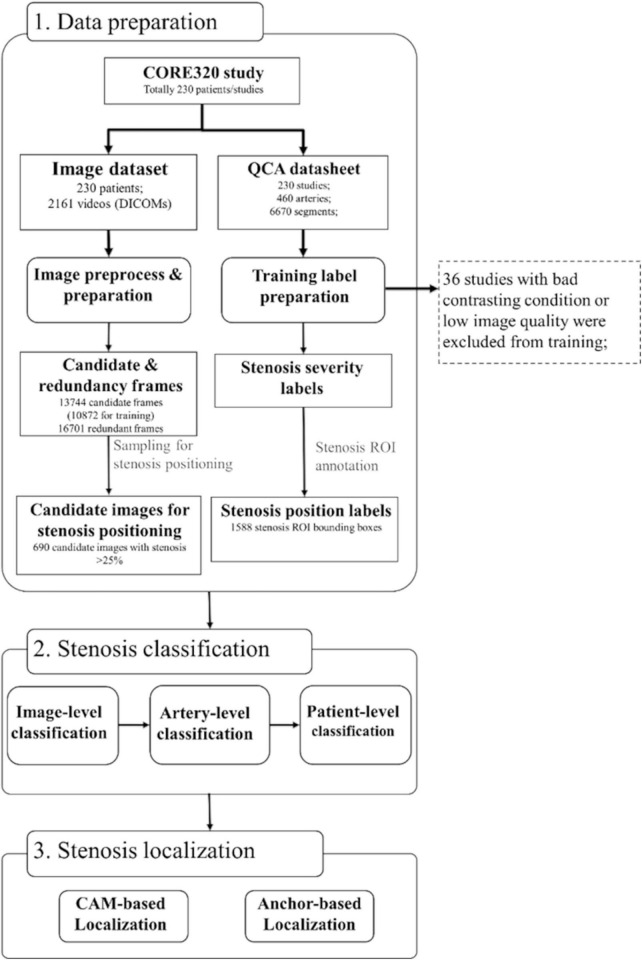
Dataset and algorithm workflow. Three steps of data preparation, stenosis classification, and stenosis positioning were presented. The steps of image and training label preparation including coronary artery selection, viewing angle selection, and contrasting frame detection were designed in a fully automatic manner. Stenosis severity classification training was performed on image-level, artery-level, and patient-level. Stenosis positioning was performed in two methods of CAM-based and anchor-based methods. CAM, class activation map; QCA, quantitative coronary angiography.

The entire study workflow is summarized in Figure 1. All the CAG studies were saved in the universal DICOM format with a resolution of 512 × 512, 15 fps, typically 60–200 frames per view. The detailed imaging parameters were summarized in Supplementary Table 1. Coronary type (left and right coronary artery, LCA, and RCA) was classified initially by experts in a small subset (19 patients). This was then leveraged by training an inception-V3 classifier (27) for automated coronary selection (100% classification accuracy was obtained). To identify the angle views of the CAG images, DICOM tags were used. Overall 4 angles for LCA [left anterior oblique (LAO) Cranial, LAO Caudal, right anterior oblique (RAO) Cranial, and RAO Caudal] and 3 angles for RCA (LAO, straight RAO, and shallow LAO/RAO Cranial) were used based on the optimal view map (OVM) (20).

A CNN + Long Short Term Memory (LSTM) network was implemented for the candidate frame selection from 19 patients (146 videos in total, and 18,688 frames overall). A candidate frame was defined as an image with good quality, full contrasting, clear vessel border, and anatomical significance of stenosis (if it had stenosis) in a video frame. Inception-v3 was employed as a basic classifier to recognize full-contrasting frames and non-contrasting frames as candidates or redundancy frames. Then, the fully connection layer of inception-v3 was output to a bi-directional LSTM with 32 time-steps (units), and also concatenated with the output of forward and backward LSTM units. The concatenation result was connected with a multi-layer perception (MLP, with one hidden layer) and a binary activation layer (sigmoid). The detailed structure of inception-v3 and LSTM is provided in Supplementary Figure 1. The inception model was initialized by ImageNet weights and then pre-trained for 200 epochs with the initial learning rate (LR) of 1e^−4^ with the loss function as binary entropy. The LSTM was initialized using Xavier uniform method for kernels and orthogonal matrix for recurrent weights, then trained for 100 epochs with LR = 4e^–5^ with the loss function of convolutional F1 score. Typically, this strategy selected 5–10 candidate frames per video.

The performance of candidate frame detection was tested with 582 videos from 175 patients using mean error and standard deviations of beginning contrasting frame (BCF) and ending contrasting frame (ECF) between ground-truth and prediction. The acceptance and error rates were also calculated with average differences of BCF and ECF in a pre-defined range (2), in which accept rate with the error ≤3 frames and error rate with the error ≥10 frames. Performance was reported using classification accuracy, F1, and Kappa.

### Stenosis classification

For the stenosis classification, the quantitative coronary angiography (QCA) results previously documented per segmental level in the CORE320 study were utilized as a reference (25, 26, 28). In the current study, in order to accommodate with our study goals (separating cases with normal coronary arteries or mild stenoses from that with higher stenosis severities), coronary stenosis severities were re-categorized into the per-coronary artery, i.e., per LCA or RCA, and grouped into three categories of < 25%, 25–99%, and total occlusion in 3-categories (CAT), or two groups of < 25 and ≥ 25% in 2-CAT. It is known that there is a mismatch between the coronary stenosis severity and functional significance. Even the intermediate stenosis lesion can present functionally significant stenosis by fractional flow reserve (29, 30). Since we aimed to develop a safety screening tool for a large volume of the CAG images, we selected a stenosis threshold with high specificity to correctly separate cases that does and does not need further functional stenosis assessment.

Different CNN architectures of ResNet-50, ResNet-101, Inception-v3 and InceptionResNet-v2 were employed for the image-level stenosis classification training and prediction. And the inception-v3 was employed finally in image-level, artery- and patient-level stenosis prediction, since it has a good balance in transfer timing, parameter size and performance. The training was performed on 4 models of LCA for each angle view and one model of RCA combining the three angle views due to the complicated features of LCA when compared to the RCA (31).

**FIGURE 2:**
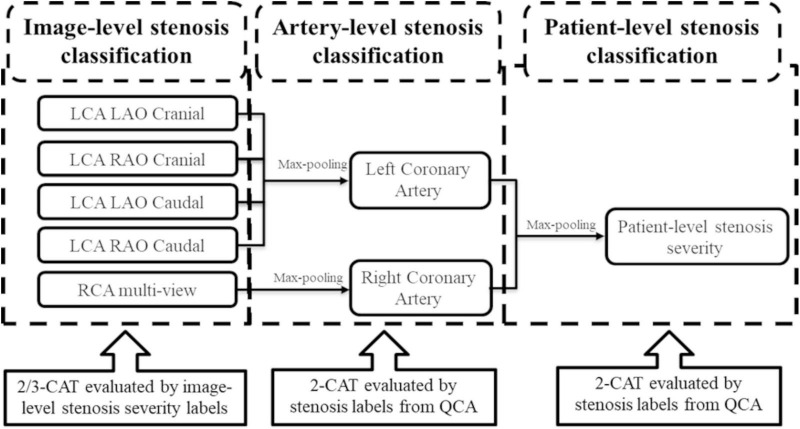
The architecture of the output of the stenosis classification inception model. A max-pooling layer was added to the output of inception to evaluate the artery-level stenosis prediction and the patient-level stenosis prediction. LCA, left coronary artery; LAO, left anterior oblique; RAO, right anterior oblique; RCA, right coronary artery; QCA, quantitative coronary angiography.

The classification prediction of artery-level and patient-level was implemented by a multi-view analyzing architecture, as described in Figure 2. For artery-level prediction, CNN scores from 4, or, 3 angle-views were combined and fed into a max pooling layer to generate LCA/RCA classification results, respectively. Similarly, the patient-level prediction scores were generated by feeding LCA and RCA scores into another max pooling layer (Figure 2). For the image-level labeling, 2 or 3-CAT stenosis categories were assigned in each angle view. For the artery-level labeling, 2-CAT stenosis categories were assigned in each coronary artery, i.e., in the LCA and the RCA. Overall 10,872 frames from 194 studies were used for image-level stenosis classification training and 13,744 frames from 230 studies were used for the fourfold cross-validation. The distribution of the cases in the image-, artery-, and patient-levels are summarized in Table 1. Performance of image-level classification on 3- CAT and 2- CAT with and without redundancy training was reported using accuracy, sensitivity, F1, Kappa, and area under the curve (AUC). Performance of artery-level and per-patient level classification was assessed on the 2-CAT with redundancy training image-level results and reported using accuracy, sensitivity, and AUC.

**TABLE 1:** Distribution of the cases in each stenosis severity category used for the image view, artery, and patient-levels validation.

Stenosis severity	Image level (LCA—LAO Cranial)	Image level (LCA—RAO Cranial)	Image level (LCA—LAO Caudal)	Image level (LCA—RAO Caudal)	Image level (RCA)	Coronary artery level (LCA)	Coronary artery level (RCA)	Per-patient level
<25%	624 (32.7%)	752 (33.0%)	673 (43.8%)	789 (34.7%)	2,292 (39.9%)	57 (24.8%)	84 (36.5%)	46 (20.0%)
25–99%	1,123 (58.9%)	1,393 (61.1%)	673 (43.8%)	1,333 (58.5%)	3,181 (55.4%)	132 (57.4%)	118 (51.3%)	127 (55.2%)
100%	160 (8.4%)	135 (6.0%)	189 (12.3%)	155 (6.8%)	272 (4.7%)	41 (17.8%)	28 (12.2%)	57 (24.8%)

Overall 13,744 frames from 230 studies were used for the image level validation. Each of the coronary artery level and per-patient level validations were performed on 230 cases.

LCA, left coronary artery; LAO, left anterior oblique; RAO, right anterior oblique; RCA, right coronary artery.

### Redundancy training

In the image-level stenosis classification training, the redundancy frames were accessorily added to the training dataset but not in the validation set. A redundancy frame was defined as a background frame without any contrasting agent in arteries. Thereafter, the redundancy categories were comprised of background frames with the roughly same amount of samples as the target categories in training dataset. Subsequently, there are 12,351 redundancy frames combined with 10,872 candidate frames in 3- and 2- CAT image-level training, namely redundancy training, as the similar methods used before (3, 32). It is expected that the use of redundancy frames can hedge against the invalid feature learning and reduce the train/test overfitting.

### Stenosis localization

For the stenosis positioning, two methods were investigated: (1) class activation map (CAM) (33) based on the back-propagation from the stenosis classification decision and (2) anchor-based FPN. The anchor-based FPN model is developed from RetinaNet (34) and FPNs (35), using the pre-trained inception-V3 as backbone. The network structure is demonstrated as Supplementary Figure 2. The 1st, 2nd, and 3rd feature map in the pyramid were derived from the output of the concatenate feature before the 1st, 2nd, and 3rd pooling layer, respectively. The 4th and 5th feature maps were down sampled from the previous layers. For FPN inputs, 1,588 positioning boxes with a minimal size of 35 × 35 pixels were annotated by two independent expert cardiologists. The shapes of anchor were preset by K-Means clustering method with seven different groups of height and width. The anchor-based model was trained with Learning Rate = 1e^–4^ over 500 epochs. Then FPN was built on the feature maps of pre-trained classification models. The same reader-annotated bounding boxes were also used for the evaluation of the CAM-based localization technique. For the positioning training and fourfold evaluation, 690 frames with > 25% stenosis were used (Figure 1).

The performances of the two stenosis localization methods were assessed by the metrics of global-sensitivity, per-stenosis-sensitivity (Sens_s), per-stenosis-specificity (Spec_s), and mean square error (MSE). Global-sensitivity was defined as the recall of localization for the most significant stenosis in the images, which is similar to AR^∧^(max = 1) in COCO benchmark (21). Sens_s and Spec_s were defined as the recall rate of all stenosis localizations in the images. MSE was assessed in 512 × 512 images for the CAM-based model and the anchor-based models. Due to the lower resolution, metrics for the CAM-based model were calculated with Intersection over Union (IoU) > 0.2 in the CAM-based model whereas IoU > 0.5 for the anchor-based model.

### Statistical analysis

All the statistical evaluation was performed in Python (version 3.6; Python Software Foundation, Wilmington, Del).^2^ In this study, the calculation for diagnostic performance was based on a per-patient approach, including image-level severity classification. Accuracy, f1-score, and Cohen’s Kappa were calculated for image-level stenosis classification; receiver operating characteristic (ROC) analysis and areas under the curves (AUC) were used to further evaluate the image-/artery-/patient-level diagnostic performance. Stenosis positioning was evaluated by sensitivity, specificity, and MSE as described above. The CNN, LSTM, CAM, and anchor-based models were performed on TensorFlow (version 2.4.0), Python (version 3.6), and the Ubuntu system (version 20.04). All metrics were computed using Scikit-learn, version 0.19.1. Continuous variables that were normally distributed were summarized and reported as means ± standard deviations.

## Results

### Patient characteristics

The study participants’ characteristics are given in Table 2. A total of 230 individuals were included in our analysis. The median age was 62 years (IQR 55, 69), 70% were men, 45% were white, 82% had hypertension, 71% had dyslipidemia, 16% were current smokers, and 27% had a high pretest probability of obstructive CAD.

**TABLE 2:** Clinical characteristics of the study participants.

Characteristic	Included (*n* = 230)
Age (years)	62 (55, 69)
Age ≥ 60 years	134 (58%)
Male sex	160 (70%)
Race	
White	103 (45%)
Black	18 (8%)
Asian	105 (46%)
Other	4 (2%)
Body mass index (BMI, kg/m^2^)	26 (24, 29)
Obesity (BMI ≥ 30 kg/m^2^)	51 (22%)
Hypertension	188 (82%)
Diabetes mellitus	80 (35%)
Dyslipidemia	159 (71%)
Current smoker	35 (16%)
Family history of CAD	88 (41%)
Diamond-forrester risk score	
Low	4 (2%)
Intermediate	164 (71%)
High	62 (27%)
Previous cerebrovascular accident	9 (4%)

A total of 230 individuals were included in our analysis. The median age was 62 years (IQR 55, 69), 70% were men, 45% were white, 82% had hypertension, 71% had dyslipidemia, 16% were current smokers, and 27% had a high pretest probability of obstructive coronary artery disease.

### Candidate frame selection

The automatic model achieved a mean error of 2.05 and 2.27 in BCF and ECF detection, respectively. The acceptance and error rates were 83% and 5.0%. A common feature of misclassified cases was a relatively short contrast duration in the video (typically < 5 frames with adequate vessel-to-background contrast). The network did not adequately handle this type of condition because the training dataset had very few instances of short-duration contrasting frames.

### Stenosis classification

**FIGURE 3:**
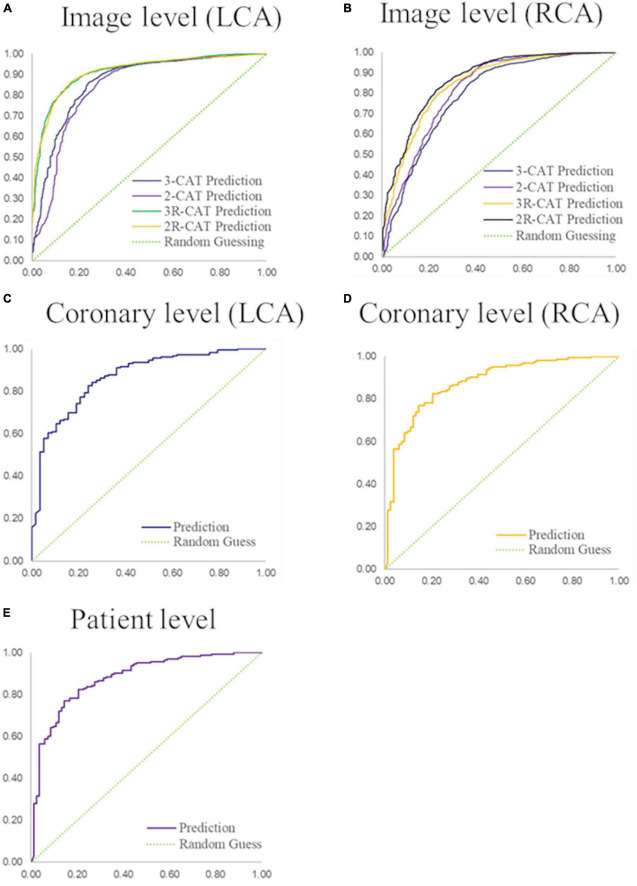
Performance of coronary stenosis classifications in image, coronary artery, and patient levels. **(A,B)** ROC curves of image-level classification on 3-CAT and 2-CAT with and without redundancy training on LCA and RCA. **(C,D)** ROC curves of coronary artery level classification on LCA and RCA. **(E)** ROC curve of patient-level classification. The AUC values are summarized in Table 3. RCA, right coronary artery; LCA, left coronary artery; AUC, area under the curve.

**FIGURE 4:**
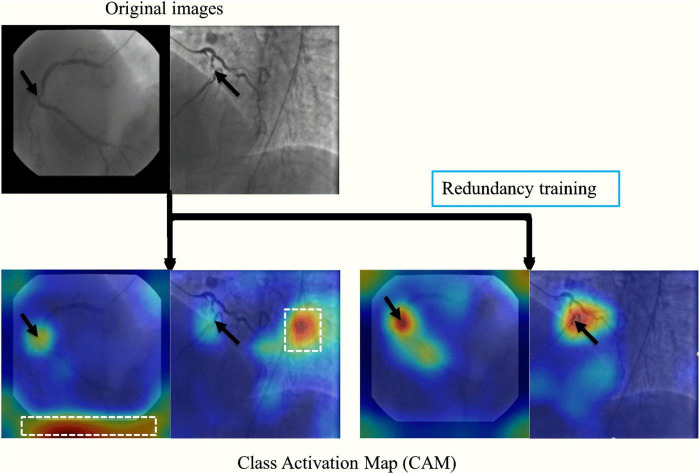
A representative image of the effect of the redundancy training demonstrated in a heatmap style. In the original training, the model had mid-to-high level attention on background regions. The redundancy training reduced the overfitting caused by background structures and improved the performance of stenosis classification.

The stenosis classification results in 3-CAT and 2-CAT with and without redundancy training models are summarized in Table 3 and Figure 3. In brief, the image-level classification performance was better in 2-CAT than 3-CAT for the LCA while not significantly different for the RCA. The redundancy training improved the AUC values for both 2-CAT and 3-CAT, as well as the accuracy, F1-score, and kappa score in 2-CAT. Based on the better performance in 2-CAT as well as our aim to separate normal coronary/mild stenoses from higher severity of stenosis, 2-CAT evaluation was performed for artery-level (LCA and RCA) and patient-level classification. The accuracies were 0.83, 0.81, 0.85, the sensitivities were 0.94, 0.90, and 0.96 and AUCs were 0.87, 0.88, and 0.86 at the artery-level; LCA and RCA, and at the per-patient level, respectively. A representative image illustrating the effect of the redundancy training is demonstrated in Figure 4 with visualization aided by a heatmap. The overfitting caused by background structures is markedly reduced, likely resulting in the improvement in classification performance.

**TABLE 3:** The image-level stenosis classification performance for the 2-category and 3-category severity levels.

	3-CAT (<25, 25–99, 100% stenosis)					2-CAT (<25, >25% stenosis)				
	**Acc.**	**Sensitivity**	**F1**	**κ**	**AUC**	**Acc.**	**Sensitivity**	**F1**	**κ**	**AUC**
LCA	0.71 ± 0.02	0.78 ± 0.04	0.65 ± 0.05	0.50 ± 0.07	0.77	0.77 ± 0.01	0.90 ± 0.06	0.71 ± 0.06	0.46 ± 0.04	0.80
LCA w/R	0.70 ± 0.06	0.72 ± 0.04	0.70 ± 0.06	0.44 ± 0.10	0.82	0.79 ± 0.02	0.89 ± 0.04	0.74 ± 0.07	0.51 ± 0.06	0.84
RCA	0.83 ± 0.02	0.82 ± 0.01	0.81 ± 0.04	0.70 ± 0.04	0.86	0.84 ± 0.01	0.92 ± 0.02	0.77 ± 0.01	0.56 ± 0.03	0.83
RCA w/R	0.77 ± 0.02	0.81 ± 0.04	0.77 ± 0.03	0.59 ± 0.04	0.90	0.83 ± 0.01	0.90 ± 0.01	0.80 ± 0.03	0.63 ± 0.03	0.89

The classification performance with and without the redundancy training is presented. The performance of image-level stenosis classification results of LCA was reported as a combined result of four angles. Performance is assessed using accuracy, sensitivity, F1-score, kappa, and AUC.

LCA, left coronary artery; RCA, right coronary artery; LCA w/R, LCA with redundancy training; RCA w/R, RCA with redundancy training; Acc., accuracy; F1, weighted F1-score; κ, Cohen’s Kappa; AUC, area under the curve.

Additionally, the image-level classification performances of different CNN models of ResNet-50, ResNet-101, Inception-v3 and InceptionResNet-v2 were compared in Table 4. The comparative result suggests that Inception-v3 is the most suitable one among all four models, because of its fast inference speed, small size, and high accuracy in many tasks.

**TABLE 4:** The comparative study of different CNN models in image-level stenosis classification performance.

Method	LCA w/R 3-CAT		RCA w/R 3-CAT		LCA w/R 2-CAT		RCA w/R 2-CAT		FLOPS	Parameter memory	Inference time
	**Acc.**	**AUC**	**Acc.**	**AUC**	**Acc.**	**AUC**	**Acc.**	**AUC**			
Inception V3	0.73	0.82	0.79	0.90	0.81	0.84	0.84	0.89	5.72 G	91 M	13.93 ms
ResNet-50	0.67	0.76	0.64	0.75	0.76	0.80	0.71	0.64	3.87 G	98 M	12.30 ms
ResNet-101	0.58	0.78	0.63	0.87	0.81	0.78	0.76	0.71	7.6 G	170 M	15.33 ms
Inception ResNetV2	0.75	0.83	0.80	0.92	0.80	0.83	0.82	0.88	13.18 G	214 M	18.16 ms

The comparative study of different CNN models is presented by the performance of image-level stenosis classification. Performance is assessed using accuracy (Acc.) and AUC (> 25% stenosis vs. < 25% stenosis). The metrics of floating-point operations (FLOPS), parameter memory (unit: byte) are referred from the following website: https://github.com/Coderx7/SimpleNet.

Acc., accuracy; LCA w/R 3-CAT, LCA with redundancy training in three category classification; RCA w/R 3-CAT, RCA with redundancy training in three category classification; LCA w/R 2-CAT, LCA with redundancy training in two category classification; RCA w/R 2-CAT, RCA with redundancy training in two category classification; AUC, area under the curve; FLOPS, floating-point Operations.

### Stenosis localization

**FIGURE 5:**
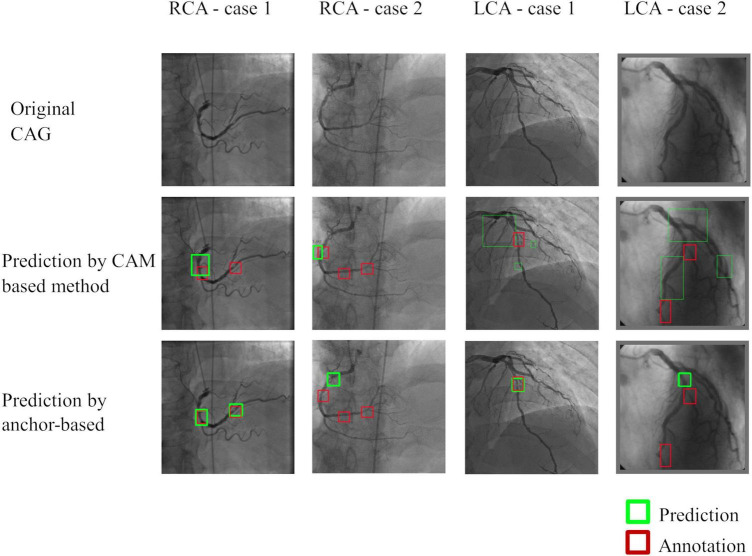
Representative images of stenosis position localization experiments. Predicted boxes from the anchor-based model produced more accurate boxes when compared to the CAM-based model. Multiple stenoses in distal coronary arteries or branches were difficult for correct localization, which was the main reason for the failed cases.

Quantitative results were summarized in Table 5. In brief, the anchor-based FPN method showed better performance than the CAM-based method by all the metrics studied. Both the localization techniques performed better for RCA images than for LCA images. In both methods, Sensitivity was low due to the many annotations that highlighted small lesions that had ambiguous feature patterns in the arteries. Performance was also lower when there were multiple stenoses in distal coronary arteries or branches (see Figure 5 for illustration).

**TABLE 5:** Performance of the stenosis localization algorithms for the LCA and RCA.

	CAM-based				Anchor-based			
	**Global-sensitivity**	**Sens*_s_***	**Spec*_s_***	**MSE (deviation)**	**Global-sensitivity**	**Sens*_s_***	**Spec*_s_***	**MSE (deviation)**
LCA	0.59	0.25	0.43	103.3 (71.18)	0.68	0.44	0.68	39.3 (40.00)
RCA	0.61	0.17	0.51	79.5 (47.21)	0.70	0.51	0.77	37.6 (51.63)

Results are presented as the global sensitivity, sensitivity, specificity, and MSE for the two techniques presented—the CAM-based model and the anchor-based model. Global-sensitivity was defined as the sensitivity of one most severe stenosis localization per image. Due to the low resolution, metrics (Sens, Sens_s, Spec_s) for the CAM-based model were calculated with IoU > 0.2 whereas the metrics for the anchor-based model were calculated with IoU > 0.5.

LCA, left coronary artery; RCA, right coronary artery; Sens_s, per-stenosis sensitivity; Spec_s, per-stenosis specificity; MSE, mean square error; CAM, class activation map; IoU, intersection over union.

## Discussion

In this study, we developed a CAG stenosis detection and localization tool to facilitate safety screening of a large volume of the CAG images. The main findings from the present study are summarized as follows: (1) the fully automatic, end-to-end workflow, which eliminated the vessel extraction and segmentation step for supervised-learning was developed; (2) the multi-view CAG analyzing architecture for artery- and patient-level stenosis classification, achieving an accuracy of 0.85, a sensitivity of 0.96 and an AUC of 0.86 at the per-patient level; (3) redundancy training improved classification performance, hedged against the invalid feature learning and reduced the error between the training and validation sets; (4) stenosis localization was investigated with two methods of CAM-based and anchor-based models, with superior quantitative results with the anchor-based models.

End-to-end workflow is advantageous in reducing human interaction steps. In our proposed workflow, once applied to the CAG videos, the model automatically selects the optimal frames, performs stenosis classification and localizes stenosis positions, providing robust results at both the artery and patient levels. Our workflow is advantageous in a large volume clinical setting or quality control purposes because the timely screening of many CAG videos to identify cases with normal or only mild stenoses consequences to secure more time on the cases with higher stenosis severities, which can translate into improved productivity and facilitated safety screening. Additionally, by providing stenosis classification and localization, the reader/physician can quickly focus on the lesion and perform quantitative CAG in an efficient manner.

The candidate frame selection performance presented here was better than previous publication by another group (4), likely due to the use of the bi-directional CNN + LSTM network to effectively extract high dimensional features of contrast flows from images, so that the network can effectively detect the changing trend in temporal sequences and find the contrast frames with higher accuracy than the RNN-only method (4). The stenosis classification results in the current study are encouraging that are comparable and sometimes outperforming when compared to the methods reported in previous studies; in which the image-based stenosis classification methods (5, 36) presented patient-level 2-CAT sensitivity of 0.80, 0.87; other three vessel- and patch-based studies (6, 9, 17) presented accuracies of 0.94, 0.97, and 0.92, respectively. We attribute our favorable results to addressing different aspects of a typical CAG study such as multiple angle views, background frames, and visually insignificant features of vessel stenoses through redundancy training to reduce overfitting in classification training.

Redundancy training is an effective tool for improving classification accuracy and reducing the error between the training and validation sets. In the original training, CNNs may be activated by invalid features such as image background and artifacts, which can be visualized from CAM heatmap. Comparatively, the redundancy frames were introduced as new categories in redundancy training, therefore the stenosis features were more activated on effective features such as vessel morphology, intensity change and narrow characteristics.

The comparison between 2-CAT and 3-CAT classification implies extra characteristics in stenoses analyzing. From the experimental result, the image-level classification performance is better in 2-CAT than 3-CAT for the LCA (accuracy = 0.77 vs. 0.71) while not significantly different for the RCA (accuracy = 0.84 vs. 0.83). One possible reason is that LCA anatomy presents itself with more variation than RCA (31), causing adverse factors against the CNN models to detect vascular blockage and occlusion in 3-CAT classification. Another explanation is that there are slightly imbalanced category distributions in 3-CAT classification than in 2-CAT, resulting in reduced accuracy (in LCA) and sensitivities (both in LCA and RCA).

Our study also explores a solution to the stenosis localization problem *via* an object detection framework. Two different stenosis localization methods of CAM and FPN were compared. The CAM-based model has the strength of employing a simple derivation that uses stenosis classification as a backbone model. However, as the activation map should be calculated by feature maps in deep layers from CNNs, CAM method is unfavorable for fine-grained and multiple object detection, such as small blood vessel stenoses in the same CAG image. In contrast, anchor-based model showed a better performance for stenosis positioning, since the different scales of features can be well exploited by feature pyramid structure. The trade-off is that the additional annotations and supervised-learning procedure were necessary for training the anchor-based model. Additionally, the comparison of the stenosis localization performances between RCA and LCA also support our viewpoint that the complexity of morphology and structure of angiographic vessels may be a severely adverse factor to the accuracy of the algorithms (classification and localization). In LCA angle views, two main arteries (LAD and LCX) may interlap on the 2-dimensional CAG image and twist with each other, raising difficulties in stenosis visualization. In some cases, there are multiple lesions in separate vessels or segments in LCA (such as second diagonal, second obtuse marginal or posterolateral), with vague and insignificant visual characteristics. By comparison, RCA has clearer vessel shapes and simpler morphologic characteristics so that more significant stenosis features. Therefore, all the above factors lead to better localization performances with both methods for RCA than for LCA.

Future work will aim at the following aspects. (1) We could perform an external validation in different studies as a means to generalize our technique and further improve performance. We believe that the proposed method will achieve good results with new images. Considering that the new dataset may have different imaging parameters (angle views, phase intervals, and FOVs), we may have to adjust the image pre-processing algorithm to accommodate the new images. Furthermore, transfer learning in a small subset could also improve performance. (2) Application in a variety of clinical or investigative scenarios beyond safety screening with different clinical goals such as fine-grained stenosis classification/localization.

Our study had a few important limitations. Training and evaluation were performed in the same cohort. A validation study using an external cohort is needed to accurately assess the performance of our techniques. Stenosis classification was simply categorized into three groups of < 25, 25–99%, and total occlusion for 3-CAT while < 25 and 25–100% stenosis for 2-CAT. Our aim was to develop a tool that identifies normal and mild stenosis cases within a large cohort. In this regard, more granular categories for mild to moderate stenosis may be considered for different clinical or investigational purposes, such as the detection of hemodynamically significant stenosis.

## Conclusion

In conclusion, a fully automatic end-to-end deep learning-based workflow for CAG images that eliminates the vessel extraction and segmentation step was accomplished. Our redundancy-based algorithm showed high accuracy for stenosis classification, and accurate localization was achieved by an anchor-based model. This end-to-end approach may facilitate safety screening in high-volume centers and in clinical trial settings.

## Data availability statement

The raw data supporting the conclusions of this article will be made available by the authors, without undue reservation.

## Ethics statement

The studies involving human participants were reviewed and approved by the Johns Hopkins Institutional Review Board. The patients/participants provided their written informed consent to participate in this study.

## Author contributions

CC and YK contributed to conception and design of the study, data analysis, manuscript drafting, and revisions. HV, MO, JL, and BA-V participated in the design and coordination of the study as well as manuscript revisions. All authors have read and approved the final manuscript.

## Footnotes

1
https://clinicaltrials.gov/ct2/show/NCT00934037

2
https://www.python.org

## Supplementary material

Supplementary Figure 1 The detailed structure of inception-v3 and LSTM. Inception-v3 was employed as a basic classifier to recognize full-contrasting frames and non-contrasting frames as candidates or redundancy frames. Then, the fully connection layer of inception-v3 was output to a bi-directional LSTM with 32 time-steps (units), and also concatenated with the output of forward and backward LSTM units. The concatenation result was connected with a multi-layer perception (MLP, with one hidden layer) and a binary activation layer (sigmoid).

Supplementary Figure 2 The architecture of the anchor-based feature pyramid network for stenosis localization. The 1st, 2nd, and 3rd feature map in the pyramid were derived from the output of the concatenate feature before the 1st, 2nd, and 3rd pooling layer, respectively. The 4th and 5th feature maps were down sampled from the previous layers. The shapes of anchor were preset by K-Means clustering method with seven different groups of height and width.
