# Supplementary material for: Deep learning-based end-to-end automated stenosis classification and localization on catheter coronary angiography
Source: Front Cardiovasc Med. 2023 Feb 7;10:944135. doi: 10.3389/fcvm.2023.944135 (PMC9941145; doi:10.3389/fcvm.2023.944135)
Supplement: Supplementary file 1 [file Table_1.DOCX]

**Supplemental Table 1.** Imaging parameters.

|  | LCA | | | | | RCA | | |
| --- | --- | --- | --- | --- | --- | --- | --- | --- |
|  | LAO Cranial | LAO Caudal | RAO Cranial | RAO Caudal | LAO | | Cranial | RAO |
| Spatial resolution (mm) | 0.2596 | 0.2596 | 0.2596 | 0.2596 | 0.2596 | | 0.2596 | 0.2596 |
| Matrix size (width×height) | 512×512 | 512×512 | 512×512 | 512×512 | 512×512 | | 512×512 | 512×512 |
| FOV (width×height, mm) | 133×133 | 133×133 | 133×133 | 133×133 | 133×133 | | 133×133 | 133×133 |
| Frame time (ms) | 66.67 | 66.67 | 66.67 | 66.67 | 66.67 | | 66.67 | 66.67 |
| Frame number | 60-200 | 60-200 | 60-200 | 60-200 | 60-200 | | 60-200 | 60-200 |
| Primary angle (degree) | 45±15 | 45±15 | -30±15 | -30±15 | 45±15 | | 0±15 | -30±15 |
| Second angle (degree) | 20±10 | -20±10 | 20±10 | -20±10 | 0±10 | | 20±10 | 0±10 |

FOV = field of view. LCA = left coronary artery. LAO = left anterior oblique. RAO = right anterior oblique. RCA = right coronary artery.
