# Supplementary figures and images for: Deep learning-based end-to-end automated stenosis classification and localization on catheter coronary angiography
Source: Front Cardiovasc Med. 2023 Feb 7;10:944135. doi: 10.3389/fcvm.2023.944135 (PMC9941145; doi:10.3389/fcvm.2023.944135)

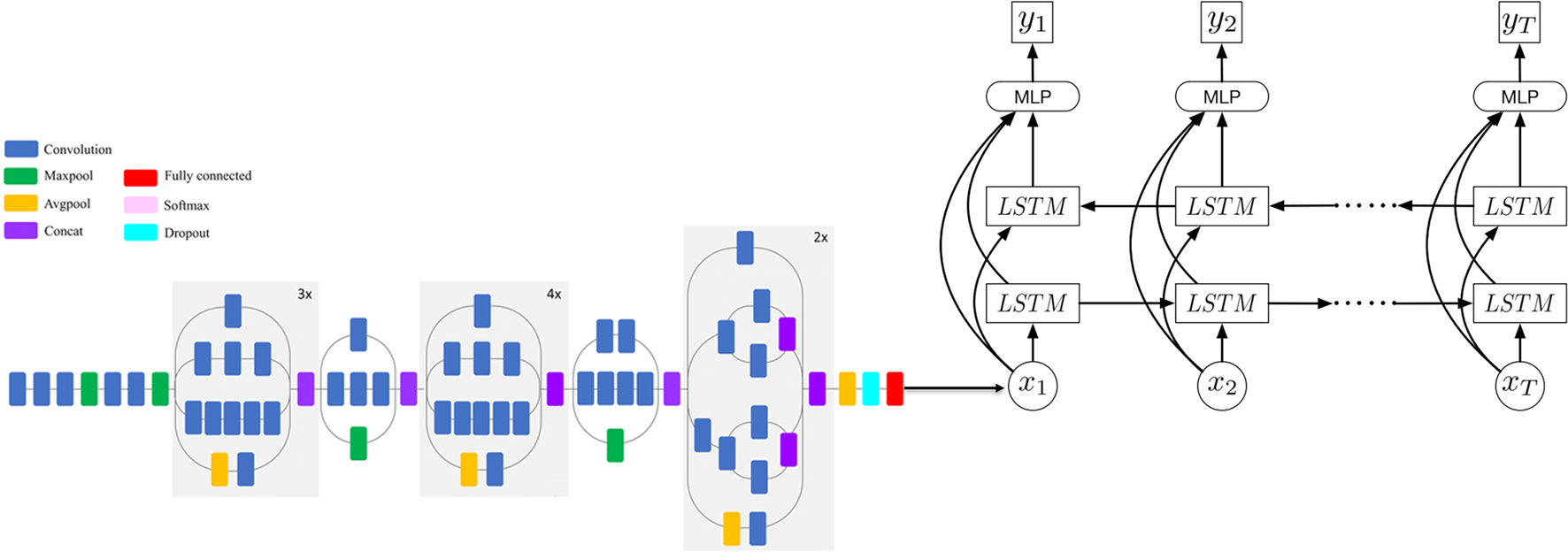

Supplement: Supplementary Figure 1 — The detailed structure of inception-v3 and LSTM. Inception-v3 was employed as a basic classifier to recognize full-contrasting frames and non-contrasting frames as candidates or redundancy frames. Then, the fully connection layer of inception-v3 was output to a bi-directional LSTM with 32 time-steps (units), and also concatenated with the output of forward and backward LSTM units. The concatenation result was connected with a multi-layer perception (MLP, with one hidden layer) and a binary activation layer (sigmoid). [file Image_1.TIF]

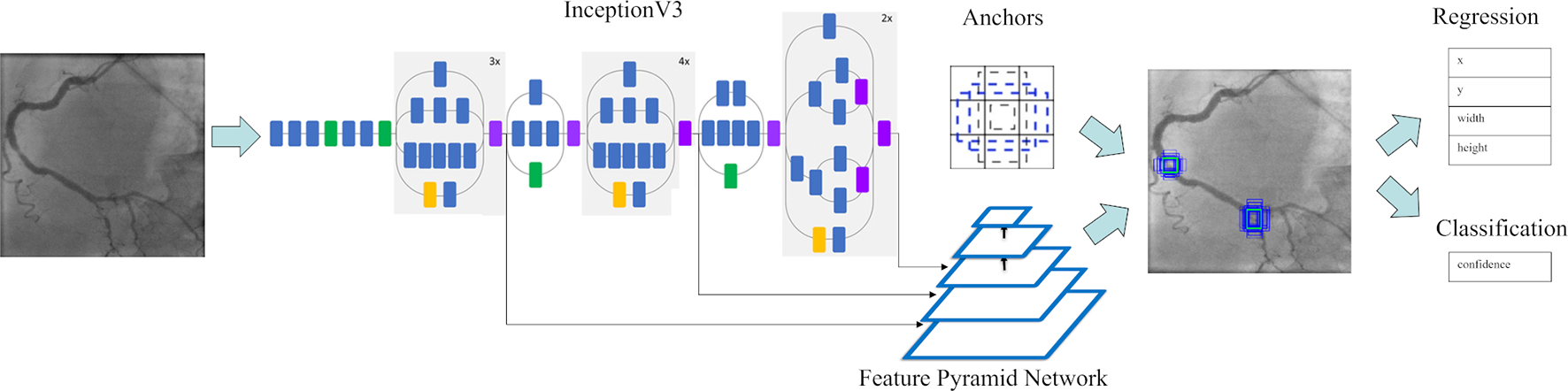

Supplement: Supplementary Figure 2 — The architecture of the anchor-based feature pyramid network for stenosis localization. The 1st, 2nd, and 3rd feature map in the pyramid were derived from the output of the concatenate feature before the 1st, 2nd, and 3rd pooling layer, respectively. The 4th and 5th feature maps were down sampled from the previous layers. The shapes of anchor were preset by K-Means clustering method with seven different groups of height and width. [file Image_2.TIF]
